# Calcium/calmodulin alleviates substrate inhibition in a strawberry UDP-glucosyltransferase involved in fruit anthocyanin biosynthesis

**DOI:** 10.1186/s12870-016-0888-z

**Published:** 2016-09-08

**Authors:** Hui Peng, Tianbao Yang, Bruce D. Whitaker, Lingfei Shangguan, Jinggui Fang

**Affiliations:** 1 Agricultural Research Service of U.S. Department of Agriculture, From the Food Quality Laboratory, Beltsville Agricultural Research Center, Beltsville, MD 20705 USA; 2 Horticulture & Landscape College, Hunan Agricultural University, Changsha, Hunan 410128 China; 3 College of Horticulture, Nanjing Agricultural University, Nanjing, Jiangsu 210095 China

**Keywords:** Fragaria vesca, Pelargonidin, Calcium signaling, UGT, Enzyme kinetics

## Abstract

**Background:**

UDP-glucosyltransferase (UGT) is a key enzyme for anthocyanin biosynthesis, which by catalyzing glycosylation of anthocyanidins increases their solubility and accumulation in plants. Previously we showed that pre-harvest spray of CaCl_2_ enhanced anthocyanin accumulation in strawberry fruit by stimulating the expression of anthocyanin structural genes including a fruit specific *FvUGT1*.

**Results:**

To further understand the regulation of anthocyanin biosynthesis, we conducted kinetic analysis of recombinant FvUGT1 on glycosylation of pelargonidin, the major anthocyanidin in strawberry fruit. At the fixed pelargonidin concentration, FvUGT1 catalyzed the sugar transfer from UDP-glucose basically following Michaelis-Menten kinetics. By contrast, at the fixed UDP-glucose concentration, pelargonidin over 150 μM exhibited marked partial substrate inhibition in an uncompetitive mode. These results suggest that the sugar acceptor at high concentration inhibits FvUGT1 activity by binding to another site in addition to the catalytic site. Furthermore, calcium/calmodulin specifically bound FvUGT1 at a site partially overlapping with the interdomain linker, and significantly relieved the substrate inhibition. In the presence of 0.1 and 0.5 μM calmodulin, *V*_max_ was increased by 71.4 and 327 %, respectively.

**Conclusions:**

FvUGT1 activity is inhibited by anthocyanidin, the sugar acceptor substrate, and calcium/calmodulin binding to FvUGT1 enhances anthocyanin accumulation via alleviation of this substrate inhibition.

**Electronic supplementary material:**

The online version of this article (doi:10.1186/s12870-016-0888-z) contains supplementary material, which is available to authorized users.

## Background

Anthocyanins, a class of phenolic compounds, function as colorful pigments in plants that attract pollinators or seed dispersers [1]. Anthocyanins also protect plants from pathogen and insect attacks as well as environmental stresses [2–4]. In addition, anthocyanins from dietary intake may contribute to the prevention of oxidative stress-mediated diseases such as cancer and inflammatory disorders [5]. Anthocyanins are synthesized from chalcones via flavonoid pathway. The last step, glycosylation of anthocyanidins, is catalyzed by a UDP-dependent glucosyltransferase (UGT) [6, 7]. Glycosylation stabilizes anthocyanidins, and thereby facilitates their transport and storage in the vacuoles. In plants there are over one hundred UGTs, which can glycosylate a variety of small molecules such as hormones, secondary metabolites and toxins. Most UGTs responsible for anthocyanin biosynthesis belong to subfamily 78 in family 1 glycosyltransferase [8]. They usually use UDP-glucose (UDP-Glc) as a sugar donor. Sugar acceptors vary depending on the plant species and organs or tissues [9, 10]. The common anthocyanidins are pelargonidin and cyanidin in strawberry fruit [11]. All the UGTs contain a conserved PSPG motif (putative secondary plant glycosyltransferase). Comparison among several crystallized UGTs in subfamily 78, such as grape VtGT1, *Medicago* MtUGT78G1 and butterfly pea Ct3GT-A indicate that they are very conserved in 3D structure. Two N- and C-terminal domains with similar Rossmann-like folds form a cleft where the substrates bind [12–14].

Anthocyanin biosynthesis is regulated by developmental and environmental signals [15, 16]. Calcium is an important second messenger in recognition of external and internal signals, and in regulation of plant growth and development as well as responses to biotic and abiotic stresses [17, 18]. Changes in cellular calcium concentration can be sensed and interpreted by calcium-sensors. Calmodulin is a ubiquitous calcium sensor to modulate the functions of its target proteins upon binding [19–21]. In *Arabidopsis*, changes in endogenous calcium levels can modulate sucrose-induced sugar uptake and regulate anthocyanin accumulation [22]. Pre-harvest spray with calcium stimulates the expression levels of the anthocyanin structural genes, such as dihydroflavonol 4-reductase (*DFR*), anthocyanidin synthase (*ANS*) and *UGT*, and increases anthocyanin accumulation in strawberry fruits [23]. Calmodulin can regulate anthocyanin accumulation in grape by affecting sucrose-induced sugar uptake [24], and activating flavonoid pathway genes [25, 26]. Changes in calmodulin abundance are correlated with the anthocyanin level in grapevine cell suspension cultures and *Alternanthera bettzickiana* seedlings [26, 27]. However, the underlying mechanism for calcium/calmodulin regulation is not clear.

Strawberry, an economically important rosaceous crop, is rich in anthocyanins [11, 28]. Commercial strawberry (*Fragaria x ananassa* Duch.) is an octoploid hybrid with a complex genetic background. In comparison, the diploid woodland strawberry (*F. vesca* L. ssp. *vesca*) has small stature, a short life cycle, and a fully sequenced genome. Thus, the woodland strawberry has been regarded as a model system for rosaceous functional genomics studies [29–31]. Recently we analyzed the expression pattern of eight UGTs (*FvUGT1*-*8*) of the subfamily 78D in different tissues of *F. vesca* [23]. *FvUGT1* was specifically expressed in fruit of the red-bearing variety, but not in those of a yellow-bearing mutant. Expression of *FvUGT1* was highly correlated with red fruit maturity, and calcium treatment stimulated its expression and anthocyanin accumulation. Thus FvUGT1 is likely the major UGT catalyzing anthocyanidin glycosylation in strawberry fruit. Here we report the biochemical characterization of anthocyanidin glycosylation by FvUGT1 and the effect of calmodulin on the enzyme’s activity.

## Results

### Structural features of FvUGT1

**Fig. 1:**
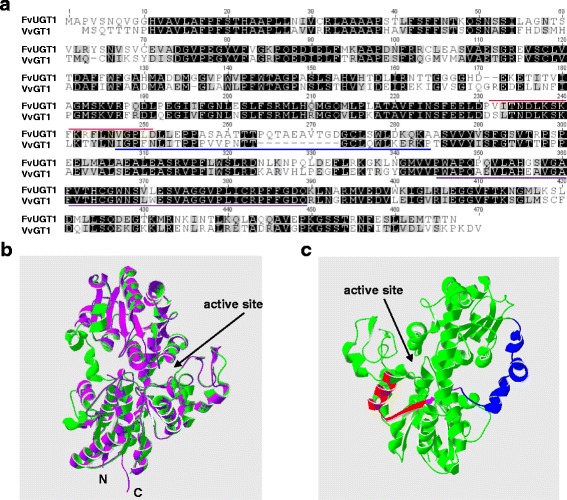
Structural features of FvUGT1. **a**. Amino acid sequence alignment of FvUGT1 and grapevine VvGT1. Identical and similar amino acids are shaded in *black* and *grey*, respectively. The putative calmodulin binding site in FvUGT1 is marked by a *red* line. The interdomain linker in VvGT1 is marked by a *blue* line. The conserved putative secondary plant glycosyltransferase (PSPG) motif is underlined with a *purple* line. **b**. Homology modeling of FvUGT1 (*green*) based on the 3D structure of VvGT1 (*purple*). **c**. Predicted 3D structure of FvUGT1 showing the regions of the putative calmodulin binding site (*red*) and the interdomain linker (*blue*). The overlapping region of the calmodulin-binding site and the interdomain linker is indicated with a *purple* line. The GenBank accession numbers and sources of proteins are FvUGT1 (KP165417; *F. vesca*), FaGT1 (AAU09442, *F*. × *ananassa*) and VtGT1 (AAB81682; grape)

The nucleotide sequence of the cloned *FvUGT1* showed the highest identity (99.4 %) to gene12538-v1.0-ab_initio, a predicted gene based on the sequenced *F. vesca* genome. The five amino acid differences between them could be the result of genome sequencing error (s) and/or misprediction. Amino acid sequences of FvUGT1 showed 96.6 and 59.8 % identity to FaGT1 from *F. x ananassa* and VvGT1 from grape, respectively [13, 32]. In particular, a conserved PSPG motif near the C-terminal portion can be easily identified by aligning with plant UGTs (Fig. 1a, Additional file 1: Figure S1). FvUGT1 also had high homology to the members of UGT78 (Additional file 1: Figure S1). Most of the characterized UGTs from this subfamily catalyze the transfer of a monosaccharide from a UDP-sugar donor to position 3 of a sugar acceptor, such as anthocyanidins and flavonols.

Further we performed homology modeling of FvUGT1 based on the 3D structure of grape VvGT1. The backbone of FvUGT1 was well matched to that of VvGT1 (Fig. 1b), suggesting that their secondary and tertiary structures are highly conserved. According to the crystal structure of VvGT1, the N- and C-terminal domains in FvUGT1 were projected to form a deep cleft and became the active site. The active site might contain two cavities which were used as binding sites for the sugar donor and acceptor [12–14]. There was an interdomain linker (aa 245–275) connecting the N-terminal and C-terminal domains. This linker among plant UGTs is highly variable with respect to length and sequence (Fig. 1 and Additional file 1: Figure S1), and is often associated with enzyme activity and domain movement [8]. These results indicate that FvUGT1 has all the structural features of UDP-Glc:anthocyanidin-3-*O*-glycosyltransferases.

### FvUGT1 is a UDP-Glc : anthocyanidin-3-*O*-glycosyltransferase

**Fig. 2:**
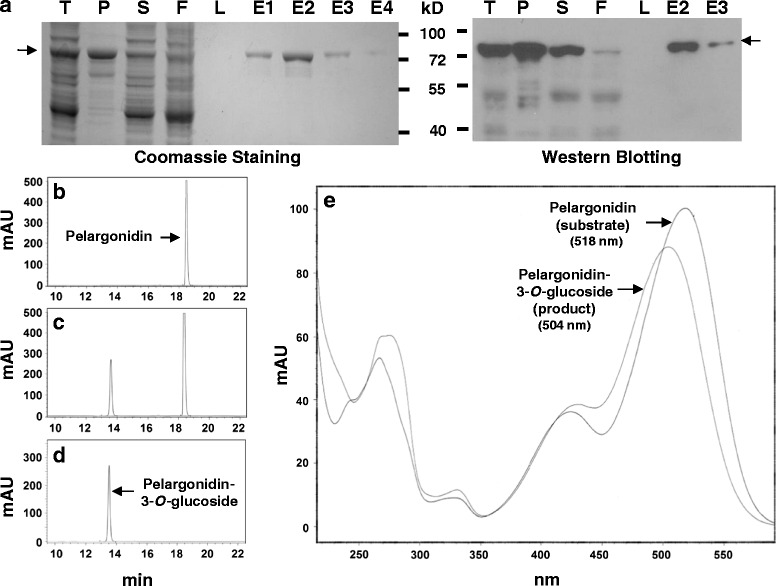
Purification and glycosylation activity analysis of recombinant FvUGT1. **a**. SDS-PAGE and Western blotting showing the purity of the recombinant FvUGT1. The SDS-PAGE gel was stained with Coomassie *Blue* (left panel). Western blotting was performed against an anti-His-tag antibody (right panel). T, total cell lysate; P, pellets; S, supernatant; F, flow-through; L, last wash; E1, 2, 3, 4, the first, second, third and fourth elution. **b**, **c**, **d**. HPLC chromatograms of pelargonidin (substrate), reaction mix of FvUGT1 glycosylation, and pelargonidin-3-*O*-glucoside (product). mAU, milli-absorbance unit. **e**. Overlaid UV absorption spectra of pelargonidin (substrate) and pelargonidin-3-*O*-glucoside (product)

To confirm that FvUGT1 has activity for anthocyanidin glycosylation, FvUGT1 fused with N- and C- terminal His-tags was heterologously expressed in *E. coli*. SDS-PAGE analysis showed that a major band with a size of 72 kD appeared in the soluble fraction. This matched the predicted size of FvUGT1-His-tag fusion protein (Fig. 2a). FvUGT1 was purified into homogeneity with a Ni-NTA column (Fig 2a), and verified by Western blot analysis against an anti-His antibody (Fig. 2a). Similarly, we also purified a His-tag protein carried in the original pET32 vector and used it as a negative control in all the experiments.

An initial glycosylation activity assay was performed using UDP-Glc and pelargonidin as substrates. HPLC-DAD analysis showed that the authentic pelargonidin and pelargonidin 3-*O*-glucoside standards had elution times of 18.5 and 13.5 min, respectively (Figs. 2b and d). The FvUGT1 catalyzed reaction produced a peak at 13.5 min (Fig. 2c) in addition to the substrate peak at 18.5 min. These two peaks were identical to those of the authentic pelargonidin and pelargonidin 3-*O*-glucoside standards. In accord with this, the UV absorption spectrum of the product peak in the FvUGT1 reaction mix closely matched that of the authentic pelargonidin 3-*O*-glucoside standard, with a maximum at 504 nm. Moreover, the UV spectrum of the FvUGT1 product differed substantially from that of the authentic pelargonidin standard, which had an absorption maximum at 518 nm (Fig. 2e). FvUGT1 was also able to use cyanidin as the sugar acceptor substrate, yielding cyanidin 3-*O*-glucoside (data not shown). The pET32 vector negative control protein carrying a His-tag did not show any glycosylation activity (data not shown). The denatured FvUGT1 did not show activity as shown by HPLC (data not shown). These results indicated that FvUGT1 is a *bona fide* UDP-Glc:anthocyanidin-3-*O*-glycosyltransferase.

### Kinetic analysis of FvUGT1 toward sugar donor substrate

**Fig. 3:**
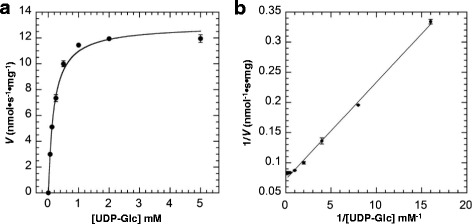
FvUGT1 kinetics toward UDP-Glc under the fixed pelargonidin (150 μM). **a**. Data were fitted to the Michaelis-Menten equation. **b**. The Lineweaver-Burk plot showing the linear relationship between 1/[pelargonidin] and 1/*V*. Each point represents the mean velocity +/−SD from triplicate determinations

To investigate the enzyme kinetics of FvUGT1, we selected the indirect Glycosyltransferase Activity assay rather than HPLC-based approach since the former approach were high-throughput yet had the comparable results to those by the latter approach [33–35]. Before determining the kinetic parameters of FvUGT1, we optimized the reaction conditions including reaction temperature and reaction time. The recombinant FvUGT1 exhibited higher activity at 37 °C than 30 °C. For the time course assay, we found that product formation showed a linear positive correlation with incubation time in the range of 10–30 min, indicating that the initial velocities were consistent within 30 min (data not shown). Thus, all initial velocity experiments were performed at 37 °C for 30 min. At the fixed concentration of pelargonidin chloride (150 μM), the resulting plot for UDP-Glc (Fig. 3a) followed the classical Michaelis-Menten kinetics (Eq. 1), as evidenced by the linear Lineweaver-Burk plot (Fig. 3b). The *V*_max_ and *K*_m_ for UDP-Glc were around 12.7 nmol · s^−1^ · mg^−1^ and 201.8 μM, respectively. However, when adding more than 150 μM pelargonidin, the *V*_max_ declined significantly although the plot still followed Michaelis-Menten kinetics (data not shown). Thus high pelargonidin could have an inhibitory effect on the enzyme activity.

### Kinetic analysis of FvUGT1 toward sugar acceptor substrate

**Fig. 4:**
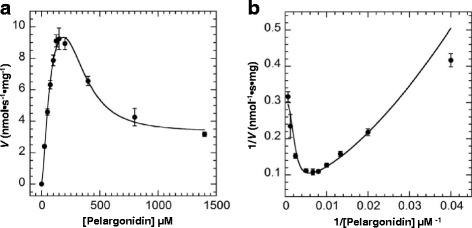
FvUGT1 kinetics toward pelargonidin under the fixed UDP-Glc (5 mM). **a**. Data were fitted to the partial uncompetitive inhibition model. **b**. The Lineweaver-Burk plot is shown to illustrate an inhibition kinetics. Each point represents the mean velocity +/−SD from triplicate determinations

Under the fixed UDP-Glc (5 mM), the enzyme activity did not show the classic Michaelis-Menten kinetics. Instead, the activity rose sharply with increasing pelargonidin up to about 100 μM, and dramatically decreased after pelargonidin exceeded 150 μM (Fig. 4a). As shown in Fig. 4b, the Lineweaver-Burk plots were non-linear. These results further support that the sugar acceptor substrate has an inhibitory effect. However, the reaction kinetics did not fit the typical substrate inhibition equation, indicating that the uncompetitive inhibition was incomplete. After fitting our data using a variety of models, we found that the kinetic of FvUGT1 fitted well to a modified Hill equation (Eq. 2) when the Hill coefficient *x* was set to 3 (Fig. 4), suggesting that FvUGT1 undergoes a partial substrate inhibition in an uncompetitive mode. Note that the *x* value was set as 3 in order to obtain the best fit to Eq. 2, suggesting that the pelargonidin might have another binding site in addition to the active site.

### Calmodulin binds to FvUGT1

**Fig. 5:**
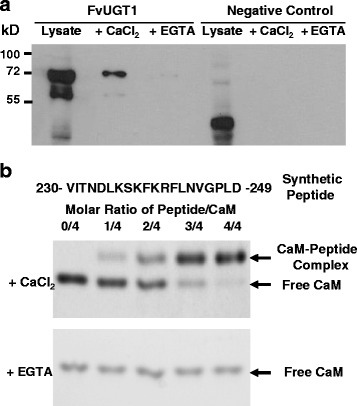
Calcium/calmodulin binds to FvUGT1 in vitro. **a**. Co-precipitation of FvUGT1 with calmodulin-agarose beads in the presence of calcium. The total proteins (lysates) from *E. coli* cells carrying either pET-FvUGT1-His-tag or negative control (pET32) were applied to calmodulin-agarose beads in the presence of 1 mM CaCl_2_ or 1 mM EGTA. Proteins bound to the beads were eluted and analyzed using Western blotting against an anti-His-tag antibody. **b**. Gel mobility shift assay showing that calcium/calmodulin binds the synthetic peptide corresponding to the putative calmodulin-binding region in FvUGT1. The synthetic peptide sequence is shown at the top of panel **b**. Arrows indicate the positions of free calmodulin and the calmodulin/peptide complex. CaM, calmodulin

Bioinformatics analysis indicated that there was a putative calmodulin-binding site (aa 230–249) in FaUGT1, since the region could form a basic amphipathic α-helix, a typical calmodulin-binding motif structure. Interestingly, the putative binding site partially overlapped with the interdomain linker (Fig. 1). To verify whether calmodulin binds FvUGT1, total bacterial proteins containing FvUGT1-His-tag were incubated with calmodulin-agarose beads in the presence of calcium or EGTA, and partitioned by SDS-PAGE. In the presence of calcium, FvUGT1-His-tag binding to the calmodulin-agarose beads was detected by Western blot analysis using anti-His antibody (Fig. 5a). By contrast, no FvUGT1 band was detected when incubation in the presence of EGTA. Calmodulin beads did not pull down anything from the bacterial cells containing a control protein (His-tag only).

Further, a gel-mobility shift assay was used to verify whether calmodulin specifically bound to the putative calmodulin-binding site (aa 230–249) in FvUGT1 (Fig. 5b). A synthetic peptide corresponding to this site on FvUGT1 was incubated with calmodulin in the presence of calcium or EGTA. After separation by native PAGE, the calmodulin-peptide complex appeared in the presence of calcium. The intensity of the complex band increased with increases in the peptide/calmodulin ratio. Only the free calmodulin band was observed after incubation in the presence of EGTA. These results demonstrated that calmodulin physically interacted with the putative FvUGT1 calmodulin binding domain in a calcium-dependent manner.

### Calmodulin increases FvUGT1 activity

**Fig. 6:**
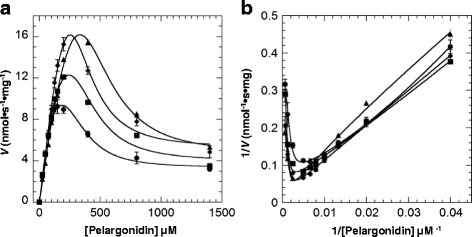
FvUGT1 kinetics showing that calmodulin can alleviate pelargonidin inhibition. **a**. Data were fitted to the partial uncompetitive inhibition model (Eq. 2). **b**. The Lineweaver-Burk plot is shown to illustrate that calmodulin did not fully relieve the inhibition kinetics. CaM, calmodulin. Each point represents the mean velocity +/−SD from triplicate determinations (●, 0 μM Calmodulin; ■, 0.1 μM Calmodulin; ♦, 0.5 μM Calmodulin; ▲, 2.5 μM Calmodulin). The concentration of UDP-Glc in the reactions was kept fixed (5 mM)

To determine the effect of calmodulin binding on enzyme activity, FvUGT1 enzymatic kinetics was further analyzed in the presence of calcium/calmodulin. Since calcium was required for coupling phosphatase activity assay, we were only able to investigate the effect of different calmodulin concentrations on FvUGT1 activity. Adding calmodulin significantly increased the enzyme activity (Fig. 6a), although the plot of velocity vs. pelargonidin concentration still exhibited a substrate inhibition mode, as evidenced by the non-linear Lineweaver-Burk plots (Fig. 6b). To obtain the best fit, the *x* value was still kept as 3, suggesting that calmodulin-binding to FvUGT1 does not affect pelargonidin binding affinity to the additional site. In the presence of 0.1, 0.5, and 2.5 μM calmodulin, the apparent *V*_max_ was increased by 71.4 %, 227 % and 246 %, respectively. These results suggest that calmodulin-binding to FvUGT1 partially relieved the substrate inhibition.

## Discussion

During anthocyanin biosynthesis, glycosylation by UGT is a critical modification because it enhances solubility and stability of the anthocyanidin aglycones and facilitates storage and accumulation in plant cell vacuoles. Often glycosylation is also one of the major factors determining natural product bioactivity and bioavailability [8–10]. Our previous study showed that the expression of *FvUGT1*, a UGT of subfamily 78D, was closely linked to anthocyanin accumulation during strawberry fruit ripening and was not expressed in an anthocyanin null mutant [23]. In this study, we found that FvUGT1 displayed the activity of glycosylating anthocyanidins and this activity was subjected to marked substrate inhibition by the sugar acceptor (e.g. pelargonidin) when supplied in excess of 150 μM. Substrate inhibition has been observed in several other characterized UGTs [36–39]. For example, soybean UGT78K1 exhibited pronounced substrate inhibition by cyanidin at 100 μM concentration [36]. Grape VtGT6 activity was inhibited when a flavonol substrate exceeded 150 μM [38]. Interestingly, the substrate displaying the inhibition effect usually is the sugar acceptor. Thus, feed-forward inhibition by the sugar acceptor might be a general phenomenon for UGTs.

FvUGT1 kinetic analysis indicated that the best model for describing the inhibition was a modified Hill equation. This equation was developed to depict a partial uncompetitive substrate inhibition model for multisubunit enzymes such as aspartate transcarbamylase and D-3 phosphoglycerate dehydrogenase [40, 41]. It has been observed that some UGTs can form homo-oligomers [42, 43]. However, it is not yet known whether FvUGT1 forms homo-oligomer. Nevertheless, based on the model, FvUGT1 may have an extra pelargonidin binding site in addition to the catalytic site. It has been suggested that the allosteric regulation exists in a rat hepatic UDP-glucuronosyltransferase [44–46] and other mammalian UGTs (reviewed in [47]). In the case of rat hepatic UGT, ATP and NADP^+^ etc. markedly reduced 4-methylumbelliferone UGT activity without competing with 4-methylumbelliferone or UDP-glucoronic acid, suggesting that the inhibitors bind to an allosteric site [44–46]. It will be interesting to investigate whether the allosteric regulation exists in FvUGT1. Moreover, FvUGT1 activity was positively regulated by calmodulin, the ubiquitous calcium sensor in plants. Calcium/calmodulin was able to bind FvUGT1 (Fig. 5a), and significantly enhanced the glycosylation activity by increasing the apparent reaction-limiting velocity at high concentrations of pelargonidin. At the same time, the *K*m value rose as the velocity was increased (Table 1), indicating that calmodulin is a *V*-type activator for FvUGT1. Although the *V*-type activation is uncommon, it has been observed in some enzymes [48–50]. For example, a chemical named Compound 14 activates GSH hydrolysis as a V-type activator [48]. For *Agrobacterium tumefaciens* ADP-Glucose Pyrophosphorylase mutants, both V_max_ and K_m_ for ATP increased in presence of activator pyruvate [50]. It is unclear how calmodulin binding causes the decrease of FvUGT1 affinity for the pelargonidin at the active site. However, calmodulin-binding did not affect pelargonidin-binding to the “allosteric site”, which suggests that they are two independent events. We noted that the calmodulin-binding site partially overlaps the interdomain linker in FvUGT1 (Fig. 1). It has been suggested that the interdomain linker is critical for regulation of UGT activity because it is involved in domain movement, activity, pocket shape, and interdomain interactions [8]. Since calmodulin usually regulates the function of its target proteins via modulation of their structural changes [19, 51], calmodulin-binding to the interdomain linker area may result in a FvUGT1 conformational change, partially relieves the substrate inhibition. Further structural characterization of FvUGT1 with its substrate complex is necessary to address whether there is another sugar acceptor binding site, where it is located, and how calmodulin relieves the substrate inhibition.

**Table 1:** Effect of calmodulin on FvUGT1 kinetic parameters toward pelargonidin

CaM (μM)	*V*_max_ (nmol · s^−1^ · mg^−1^)	*K*_m_ (μM)	*K*_i_ (μM)	*V*_i_ (nmol · s^−1^ · mg^−1^)	*n*	*r* ^2^
0	12.6 ± 0.95^a^	70.1 ± 3.0^a^	331.5 ± 13.3^b^	3.3 ± 0.35^a^	1.5 ± 0.12^a^	0.99
0.1	21.6 ± 1.88^b^	147.4 ± 20.9^b^	359.6 ± 22.7^bc^	4.0 ± 0.22^b^	1.2 ± 0.05^b^	0.99
0.5	41.2 ± 3.0^c^	288.2 ± 20.4^c^	294.3 ± 18.5^a^	5.0 ± 0.48^c^	1.2 ± 0.10^b^	0.99
2.5	43.6 ± 2.2^c^	403.5 ± 29.8^d^	386.4 ± 20.0^c^	4.7 ± 0.18^c^	1.1 ± 0.01^b^	0.99

Note: The reaction mix included 5 mM UDP-Glc, fixed concentrations of calmodulin (01, 0.5 and 2.5 μM, respectively), and 0–1,400 μM pelargonidin chloride. Different letters (a, b, c, d) in all the parameters indicate significant differences among mean values (*P* < 0.05; *t*-test). The results are based on at least three repeats in three independent experiments. CaM: calmodulin; *V*_max_: the maximal reaction rate; *K*_m_: the substrate concentration at which the reaction rate is half of *V*_max_; *K*_i_: the inhibition constant which is the concentration of inhibitor required to decrease the maximal rate of the reaction to half of the uninhibited value; *V*_i_: the reaction velocity in the presence of inhibition; *n*, *x*: Hill coefficient

Anthocyanin biosynthesis is a part of flavonoid pathway, which in turn is a major branch of the phenylpropanoid pathway [7]. Recently, a considerable amount of information has been gathered on the transcriptional regulation of anthocyanin regulatory genes and structural genes in the pathways by developmental and environmental cues, such as phytohormones, light, and sucrose [15, 16]. Calcium/calmodulin is known to be involved in the signal transduction pathways that mediate responses to environmental and hormonal stimuli [18, 51–53]. It has been shown that calcium/calmodulin regulates sucrose-induced sugar uptake [24], and the expression of flavonoid pathway genes [25, 26]. Our recent studies indicate that the foliar calcium spray on strawberry can boost the anthocyanin accumulation in fruit by stimulating the expression of several anthocyanin structural genes including *FvUGT1* [23]. This study reveals another calcium regulation of anthocyanin biosynthesis through calmodulin-binding to FvUGT1 and consequent alleviation of substrate inhibition. Alignment of the calmodulin-binding site of FvUGT1 with those in FaGT1 and grape VvGT1 showed high similarity (Additional file 1: Figure S1). Comparison among other FvUGT1 orthologs in many other plant species revealed that the region nearby the interdomain linker is also conserved in their sencondary structure, although their primary structure (aa sequences) does not show high homology (data not shown). It has been recognized that the calmodulin-binding motifs in the target proteins are not conserved. However, the target peptides usually form a basic amphipathic α-helix [20, 54]. Hence the interdomain linker area in other UGT orthologs might contain a calmodulin target site. Calcium/calmodulin may exert similar regulation of anthocyanin/flavonoid glycosylation in other plants.

## Conclusions

FvUGT1 is a key enzyme for glycosylation of anthocyanidins to yield anthocyanins in strawberry. FvUGT1 activity is subject to both negative and positive regulations. The negative regulation results from feed-forward inhibition by the sugar acceptor (anthocyanidin aglycone) at high concentration. Positive regulation is exerted by calcium/calmodulin-binding, which alleviates substrate inhibition. It is well known that several classes of flavonoids are synthesized in the flavonoid pathway, such as anthocyanins, flavonol glycosides, and proanthocyanidins. All of them compete for the same carbon source. Therefore the sophisticated regulation by calcium may allow plants to coordinate the biosynthesis of anthocyanins, flavonols and proanthocyanidins, etc. in response to environmental and developmental signals. In addition, identification of the calmodulin-binding site in FvUGT1 provides an opportunity to modify the structure/function of FvUGT1 by altering the calmodulin binding site [55]. Du and Poovaiah (2005) produced plants with different height by mutating one or two amino acids in the calmodulin-binding site of DWF1, a gene responsible for brassinosteroid biosynthesis [56]. Further structural and functional characterization of FvUGT1 with site-directed mutation analysis in the calmodulin-binding site will shed light on the importance of calcium/calmodulin regulation of anthocyanin biosynthesis in plants and the future potential for metabolic engineering of anthocyanin accumulation.

## Methods

### Plant materials

Diploid woodland strawberry (*F. vesca* ssp. *vesca*) 7th generation inbred line Ruegen F7-4 (RF7-4) was obtained from Dr. Slovin [11]. The plants were grown in a greenhouse at 26 °C with a diurnal cycle of 16 h light and 8 h darkness following normal cultivation practices. Fruit samples were collected from 4 to 5 individual plants at ripe stage (receptacles showing fully red). After harvest, the fruits were rinsed with distilled water, immediately frozen in liquid nitrogen, and kept at−80 °C for future use.

### Cloning of FvUGT1 and bioinformatics analysis

Total RNA was extracted from RF7–4 ripe fruits using the RNeasy Plant Mini Kit (Qiagen, Germantown, MD, USA) following the manufacturer’s instruction. The *FvUGT1* full-length open reading frame was amplified by the *Pfx* DNA Polymerase (Invitrogen, Frederick, MD, USA) using the gene-specific primer pair (ATGGCACCAGTATCAAACCAG/ATTGGTTGTGGTCATTTCCAAC) based on the strawberry genome data in GDR (https://www.rosaceae.org/). The FvUGT1 amino acid sequence was aligned with other plant UGTs using CLC Genomics software (CLC, Aarhus, Denmark). The calmodulin-binding site was predicted utilizing the Calmodulin Target Database (http://calcium.uhnres.utoronto.ca). The homology model of FvUGT1 was constructed based on the crystal structure of grape VvGT1 (pdb code: 2C1Z) in Swiss-model (http://swissmodel.expasy.org) [13]. The model and template were compared using the Swiss PDB viewer (http://spdbv.vital-it.ch/) [57].

### Expression and purification of FvUGT1 protein

The cDNA fragment of *FvUGT1* was subcloned into *Kpn*I and *Bam*HI sites of pET-32 (Novagen, Madison, WI, USA) in frame with both N- and C-terminal His-tags. The verified construct was transformed into *E. coli* BL21 (DE3) pLysS cells. Biosynthesis of recombinant protein was induced by adding 0.1 mM isopropyl β-D-1- thiogalactopyranoside (IPTG), and purified by Ni-NTA Superflow resins (Qiagen, Germantown, MD, USA) following the manufacturer’s instructions. The presence of recombinant protein was confirmed by SDS-PAGE and Western Blot analysis against an anti-His antibody (Novagen, Madison, WI, USA). The recombinant proteins from Fraction 2, 3 and 4 were desalted using Microcon YM-50 columns (Millipore, Billerica, MA, USA) and concentrated in 1 μg/μl with glycosylation activity test buffer (25 mM Tris–HCl, 10 mM CaCl_2_, pH 7.5). Protein concentration was measured using Bradford protein assay reagent (Bio-Rad, Hercules, CA, USA). The recombinant proteins from the same batch were aliquoted and stored at−80 °C until further use.

### HPLC-DAD analysis of anthocyanins

HPLC-DAD analysis of anthocyanidin glycosylation yielding anthocyanins was performed using an Agilent 1100 Series system (Agilent, Technologies, Wilmington, DE, USA) as previously described [23]. Identification of compounds was based primarily on comparison of HPLC elution times and absorbance spectra (200–650 nm) with those of the following authentic standards purchased from Sigma (St. Louis, MO, USA): pelargonidin, cyanidin, pelargonidin-3-*O*-glucoside and cyanidin-3-*O*-glucoside.

### Calmodulin-binding precipitation

Calmodulin-binding precipitation was performed as described with minor modification [58]. Briefly, *E. coli* cells overexpressing His-tagged FvUGT1 were lysed in the precipitation buffer containing 20 mM Tris–HCl (pH 7.5), 150 mM NaCl, 1 % Triton-X-100 and protease inhibitor cocktail (Invitrogen, Frederick, MD, USA). After centrifugation at 20,000 *g* and 4 °C, the supernatant was separated into two tubes containing either 1 mM CaCl_2_ or 1 mM ethylene glycol tetraacetic acid (EGTA), a calcium chelator (Sigma, St. Louis, MO, USA), and incubated with Calmodulin-Separopore 4B beads (Bioworld, Atlanta, GA, USA) with rotary shaking at 125 rpm and 4 °C for 1 h. The beads were spun down at 750 *g* and then washed twice with precipitation buffer plus either 1 mM CaCl_2_ or 1 mM EGTA. Proteins bound to the beads were released by adding protein loading buffer and boiling for 3 min, followed by Western blot analysis using an anti-His antibody (Sigma, St. Louis, MO, USA).

### Calmodulin-binding mobility shift assay

The peptide corresponding to the putative calmodulin-binding site in FvUGT1 (aa 230–249) was synthesized by Genemed Synthesis, Inc. (San Antonio, TX, USA). Mixes (total volume 30 μL) containing 240 pmol (4 μg) of bovine calmodulin (Sigma, St. Louis, MO, USA) and different amounts of purified synthetic peptide in 100 mm Tris–HCl (pH 7.2), plus either 1 mM CaCl_2_ or 1 mM EGTA, were incubated for 1 h at room temperature. The mixes were analyzed by nondenaturing PAGE as described [59].

### Enzyme activity assays

FvUGT1 activity was assayed using the Glycosyltransferase Activity Kit (R&D Systems, Minneapolis, MN, USA) following the manufacturer’s instructions with modification. UDP-Glc and pelargonidin or cyanidin (Sigma, St Louis, MO, USA) were selected as the glucose donor and acceptor substrates, respectively. During the glycosylation reaction, a glucose moiety was transferred from UDP-Glc to an anthocyanidin and free UDP was released. Subsequently, inorganic phosphate was removed from free UDP by a specific phosphatase and quantified using Malachite Green phosphate detecting reagents. Both glycosylation and phosphatase reactions were carried out in reaction buffer (25 mM Tris–HCl, 10 mM CaCl_2_, pH 7.5) in a 96-well microplate. To prepare the sugar donor substrate stock (100 mM UDP-Glc), 566.3 mg of UDP-Glc was dissolved in 10 ml water. For the sugar acceptor substrate stock (16.3 mM pelargonidin), we dissolved 5 mg pelargonidin chloride into 0.25 ml ethanol, and then diluted to 1 ml with H_2_O. 0.2 μg of FvUGT1 protein were used for each reaction with a final volume of 50 μl. To avoid underestimating V_max_, we set one substrate (either UDP-Glc, the sugar donor or pelargonidin chloride, the sugar acceptor) fixed with well saturated concentration. Furthermore, to ensure that we can obtain appropriate velocities to determine the kinetic parameters, we measured the initial velocity at each concentration of another substrate at several time points (15, 30 and 40 min) at 30 and 37 °C. The optimal reaction temperature was 37 °C. The initial velocities at 15 and 30 min point were in the linear range of product formation at each concentration of the substrate. Hence we selected 37 °C and 30 min as the reaction condition. To assess the effect of calmodulin, different concentrations of bovine calmodulin (Sigma, St Louis, MO, USA) were added into the reaction mix containing 5 mM UDP-Glc and 0–1,400 μM pelargonidin chloride. Wells containing all other components except for FvUGT1 served as blank controls. The color reaction was started by adding Malachite reagent A and B to each well, and read at 620 nm with DTX880 Microplate Reader (Beckman Coulter, Pasadena, CA, USA). A phosphate standard curve was used to determine the conversion factor between 620 nm absorbance and inorganic phosphate concentration.

### Enzyme kinetic analysis

Normal kinetics data were fitted to Michaelis-Menten equation (Eq. 1).

1 $$ v=\frac{V_{\max}\left[S\right]}{K_{\mathrm{m}}+\left[S\right]} $$

where [S] is concentration of the varied substrate, *V*_max_ represents the maximal reaction rate, and *K*_m_ is the substrate concentration at which the reaction rate is half of *V*_max_.

Atypical substrate inhibition data were analyzed by a partial uncompetitive inhibition model (Eq. 2) as described [40, 41, 60]:

2 $$ v=\frac{V_{\max }+{V}_{\mathrm{i}}\left(\frac{{\left[S\right]}^x}{{K_{\mathrm{i}}}^x}\right)}{1+\left(\frac{{K_m}^n}{{\left[S\right]}^n}\right)+\left(\frac{{\left[S\right]}^x}{{K_{\mathrm{i}}}^x}\right)} $$

Eq. 2 is a modified Hill Equation, where *V*_i_ is the reaction velocity in the presence of inhibition, *K*_i_ is the inhibition constant which is the concentration of inhibitor required to decrease the maximal rate of the reaction to half of the uninhibited value, *n* is a Hill coefficient, and *x* is another Hill coefficient that allows for the possibility that binding of substrate in the inhibitory mode may also be cooperative. To obtain convergence for Eq. 2, the value of *x* was fixed to an integral number, which was determined empirically to give a best fit (lowest variance).

All data were analyzed with KaleidoGraph version 4.5 from Synergy Software (Eden Prairie, MN, USA). The kinetic parameters were derived from at least three repeats. Statistic analysis of the parameters was performed using Student’s *t*-test (*P*_0.05_).

### Availability of supporting data

The data supporting the findings of this article are included within the article and in the additional files.

## Additional file

Additional file 1:Figure S1. Amino acid sequence alignment of FvUGT1 and six other plant UGTs. Color and intensity change indicates the differences in the level of conservation. Dark red and dark blue represent the highest and lowest conserved levels, respectively. α-helices and β-strands are marked by black lines. The putative secondary plant glycosyltransferase (PSPG) motif is underlined by a red line and 10 conserved sugar donor interacting residues of the PSPG motif are marked with black solid triangles. The putative calmodulin-binding region in FvUGT1 is indicated by a black open box. The GenBank accession numbers or sources of proteins are FvUGT1 (KP165417; *F. vesca*), VtGT1 (AAB81682; grape), FaGT1 (AAU09442; *F*. × *ananassa*), Ct3GT (BAF49297; *C. ternatea*), MtUGT78G1 (A6XNC6.1; *M. truncatula*), AtUGT72B1 (Q9M156.1; *A. thialiana*), MtUGT71G1 (AAW56092.1; *M. truncatula*), and MtUGT85H2 (2PQ6_A; *M. truncatula*). (PDF 787 kb)

## Abbreviations

ANS: Anthocyanidin synthase
DFR: Dihydroflavonol 4-reductase
PSPG: Putative secondary plant glycosyltransferase
UDP-Glc: UDP-glucose
UGT: UDP-glucosyltransferase

## References

[1] Winkel-Shirley B (2001). Flavonoid biosynthesis. A colorful model for genetics, biochemistry, cell biology, and biotechnology. Plant Physiol.

[2] Berli FJ, Moreno D, Piccoli P, Hespanhol-Viana L, Silva MF, Bressan-Smith R, Cavagnaro JB, Bottini R (2010). Abscisic acid is involved in the response of grape (*Vitis vinifera* L.) cv. Malbec leaf tissues to ultraviolet-B radiation by enhancing ultraviolet-absorbing compounds, antioxidant enzymes and membrane sterols. Plant Cell Environ.

[3] Li JY, Oulee TM, Raba R, Amundson RG, Last RL (1993). Arabidopsis flavonoid mutants are hypersensitive to UV-B irradiation. Plant Cell.

[4] Mohanta TK, Occhipinti A, Zebelo SA, Foti M, Fliegmann J, Bossi S, Maffei ME, Bertea CM (2012). *Ginkgo biloba* responds to herbivory by activating early signaling and direct defenses. Plos One.

[5] He J, Giusti MM (2010). Anthocyanins: natural colorants with health-promoting properties. Annu Rev Food Sci Technol.

[6] Grotewold E (2006). The genetics and biochemistry of floral pigments. Annu Rev Plant Biol.

[7] Vogt T (2010). Phenylpropanoid biosynthesis. Mol Plant.

[8] Osmani SA, Bak S, Moller BL (2009). Substrate specificity of plant UDP-dependent glycosyltransferases predicted from crystal structures and homology modeling. Phytochemistry.

[9] Yonekura-Sakakibara K, Hanada K (2011). An evolutionary view of functional diversity in family 1 glycosyltransferases. Plant J.

[10] Bowles D, Lim EK, Poppenberger B, Vaistij FE (2006). Glycosyltransferases of lipophilic small molecules. Annu Rev Plant Biol.

[11] Sun JH, Liu XJ, Yang TB, Slovin J, Chen P (2014). Profiling polyphenols of two diploid strawberry (Fragaria vesca) inbred lines using UHPLC-HRMSn. Food Chem.

[12] Hiromoto T, Honjo E, Tamada T, Noda N, Kazuma K, Suzuki M, Kuroki R (2013). Crystal structure of UDP-glucose: anthocyanidin 3-*O*-glucosyltransferase from *Clitoria ternatea*. J Synchrotron Radiat.

[13] Offen W, Martinez-Fleites C, Yang M, Kiat-Lim E, Davis BG, Tarling CA, Ford CM, Bowles DJ, Davies GJ (2006). Structure of a flavonoid glucosyltransferase reveals the basis for plant natural product modification. Embo J.

[14] Wang XQ (2009). Structure, mechanism and engineering of plant natural product glycosyltransferases. Febs Lett.

[15] Jaakola L (2013). New insights into the regulation of anthocyanin biosynthesis in fruits. Trends Plant Sci.

[16] Carbone F, Preuss A, De Vos RCH, D’Amico E, Perrotta G, Bovy AG, Martens S, Rosati C (2009). Developmental, genetic and environmental factors affect the expression of flavonoid genes, enzymes and metabolites in strawberry fruits. Plant Cell Environ.

[17] DeFalco TA, Bender KW, Snedden WA (2010). Breaking the code: Ca^2+^ sensors in plant signalling. Biochem J.

[18] Reddy ASN, Ali GS, Celesnik H, Day IS (2011). Coping with stresses: Roles of calcium-and calcium/calmodulin-regulated gene expression. Plant Cell.

[19] Bouche N, Yellin A, Snedden WA, Fromm H (2005). Plant-specific calmodulin-binding proteins. Annu Rev Plant Biol.

[20] Poovaiah BW, Du LQ, Wang HZ, Yang TB (2013). Recent Advances in Calcium/Calmodulin-Mediated Signaling with an Emphasis on Plant-Microbe Interactions. Plant Physiol.

[21] Perochon A, Aldon D, Galaud JP, Ranty B (2011). Calmodulin and calmodulin-like proteins in plant calcium signaling. Biochimie.

[22] Shin DH, Choi MG, Lee HK, Cho M, Choi SB, Choi G, Park YI (2013). Calcium dependent sucrose uptake links sugar signaling to anthocyanin biosynthesis in Arabidopsis. Biochem Bioph Res Co.

[23] Xu W, Peng H, Yang T, Whitaker B, Huang L, Sun J, Chen P (2014). Effect of calcium on strawberry fruit flavonoid pathway gene expression and anthocyanin accumulation. Plant Physiol Biochem.

[24] Lecourieux F, Kappel C, Lecourieux D, Serrano A, Torres E, Arce-Johnson P, Delrot S (2014). An update on sugar transport and signalling in grapevine. J Exp Bot.

[25] Gollop R, Even S, Colova-Tsolova V, Perl A (2002). Expression of the grape dihydroflavonol reductase gene and analysis of its promoter region. J Exp Bot.

[26] Vitrac X, Larronde F, Krisa S, Decendit A, Deffieux G, Merillon JM (2000). Sugar sensing and Ca^2+^-calmodulin requirement in *Vitis vinifera* cells producing anthocyanins. Phytochemistry.

[27] Chang-Quan W, Ye-Feng Z, Tao L (2005). Activity changes of calmodulin and Ca^2+^-ATPase during low-temperature-induced anthocyanin accumulation in *Alternanthera bettzickiana*. Physiol Plantarum.

[28] Giampieri F, Tulipani S, Alvarez-Suarez JM, Quiles JL, Mezzetti B, Battino M (2012). The strawberry: Composition, nutritional quality, and impact on human health. Nutrition.

[29] Shulaev V, Sargent DJ, Crowhurst RN, Mockler TC, Folkerts O, Delcher AL, Jaiswal P, Mockaitis K, Liston A, Mane SP (2011). The genome of woodland strawberry (*Fragaria vesca*). Nat Genet.

[30] Slovin JP, Schmitt K, Folta KM (2009). An inbred line of the diploid strawberry *Fragaria vesca* f. *semperflorens* for genomic and molecular genetic studies in the *Rosaceae*. Plant Methods.

[31] Kim SH, Shin DH, Choi IG, Schulze-Gahmen U, Chen S, Kim R (2003). Structure-based functional inference in structural genomics. J Struct Funct Genomics.

[32] Griesser M, Hoffmann T, Bellido ML, Rosati C, Fink B, Kurtzer R, Aharoni A, Munoz-Blanco J, Schwab W (2008). Redirection of flavonoid biosynthesis through the down-regulation of an anthocyanidin glucosyltransferase in ripening strawberry fruit. Plant Physiol.

[33] D’Urzo N, Malito E, Biancucci M, Bottomley MJ, Maione D, Scarselli M, Martinelli M (2012). The structure of Clostridium difficile toxin A glucosyltransferase domain bound to Mn2+ and UDP provides insights into glucosyltransferase activity and product release. FEBS J.

[34] Jones MB, Oswald DM, Joshi S, Whiteheart SW, Orlando R, Cobb BA (2016). B-cell-independent sialylation of IgG. Proc Natl Acad Sci U S A.

[35] Wu ZL, Ethen CM, Prather B, Machacek M, Jiang W (2011). Universal phosphatase-coupled glycosyltransferase assay. Glycobiology.

[36] Kovinich N, Saleem A, Arnason JT, Miki B (2010). Functional characterization of a UDP-glucose:flavonoid 3-O-glucosyltransferase from the seed coat of black soybean (*Glycine max* (L.) Merr.). Phytochemistry.

[37] Nagatoshi M, Terasaka K, Owaki M, Sota M, Inukai T, Nagatsu A, Mizukami H (2012). UGT75L6 and UGT94E5 mediate sequential glucosylation of crocetin to crocin in *Gardenia jasminoides*. Febs Lett.

[38] Ono E, Homma Y, Horikawa M, Kunikane-Doi S, Imai H, Takahashi S, Kawai Y, Ishiguro M, Fukui Y, Nakayama T (2010). Functional differentiation of the glycosyltransferases that contribute to the chemical diversity of bioactive flavonol glycosides in grapevines (*Vitis vinifera*). Plant Cell.

[39] Saleh NAM, Poulton JE, Grisebach H (1976). UDP-glucose-cyanidin 3-*O*-glucosyltransferase from red cabbage seedlings. Phytochemistry.

[40] Burton RL, Chen S, Xu XL, Grant GA (2007). A novel mechanism for substrate inhibition in Mycobacterium tuberculosis D-3-phosphoglycerate dehydrogenase. J Biol Chem.

[41] LiCata VJ, Allewell NM (1997). Is substrate inhibition a consequence of allostery in aspartate transcarbamylase?. Biophys Chem.

[42] Hashimoto K, Madej T, Bryant SH, Panchenko AR (2010). Functional states of homooligomers: Insights from the evolution of glycosyltransferases. J Mol Biol.

[43] Seko A (2006). Complex formation of glycosyltransferases and their biological significance. Trends Glycosci Glyc.

[44] Ishii Y, An K, Nishimura Y, Yamada H (2012). ATP Serves as an Endogenous Inhibitor of UDP-Glucuronosyltransferase (UGT): A New Insight into the Latency of UGT. Drug Metab Dispos.

[45] Nishimura Y, Maeda S, Ikushiro S, Mackenzie PI, Ishii Y, Yamada H (2007). Inhibitory effects of adenine nucleotides and related substances on UDP-glucuronosyltransferase: Structure-effect relationships and evidence for an allosteric mechanism. Bba-Gen Subjects.

[46] Bruni S, Chang TMS (1999). Kinetic studies of hepatocyte UDP-glucuronosyltransferase: Evidence of an allosteric enzyme. Artif Cell Blood Sub.

[47] Riches Z, Collier AC (2015). Posttranscriptional regulation of uridine diphosphate glucuronosyltransferases. Expert Opin Drug Met.

[48] Wickham S, Regan N, West MB, Thai J, Cook PF, Terzyan SS, Li PK, Hanigan MH (2013). Inhibition of human gamma-glutamyl transpeptidase: development of more potent, physiologically relevant, uncompetitive inhibitors. Biochem J.

[49] Kruep DA, Dunaway GA (1984). Properties of the Phosphofructokinase Regulatory Factors. Arch Biochem Biophys.

[50] Gomez-Casati DF, Igarashi RY, Berger CN, Brandt ME, Iglesias AA, Meyer CR (2001). Identification of functionally important amino-terminal arginines of Agrobacterium tumefaciens ADP-glucose pyrophosphorylase by alanine scanning mutagenesis. Biochemistry-Us.

[51] Yang TB, Poovaiah BW (2003). Calcium/calmodulin-mediated signal network in plants. Trends Plant Sci.

[52] Yang T, Peng H, Bauchan G (2014). Functional analysis of tomato calmodulin gene family during fruit development and ripening. Horticulture Res.

[53] Peng H, Yang T, Jurick W (2014). Calmodulin gene expression in response to mechanical wounding and *Botrytis cinerea* infection in tomato fruit. Plants.

[54] Hoeflich KP, Ikura M (2002). Calmodulin in action: Diversity in target recognition and activation mechanisms. Cell.

[55] Yang TB, Du LQ, Poovaiah BW (2007). Concept of redesigning proteins by manipulating calcium/calmodulin-binding domains to engineer plants with altered traits. Funct Plant Biol.

[56] Du LQ, Poovaiah BW (2005). Ca^2+^/calmodulin is critical for brassinosteroid biosynthesis and plant growth. Nature.

[57] Kiefer F, Arnold K, Kunzli M, Bordoli L, Schwede T (2009). The SWISS-MODEL Repository and associated resources. Nucleic Acids Res.

[58] Patel-King RS, Gorbatyuk O, Takebe S, King SM (2004). Flagellar radial spokes contain a Ca^2+^-stimulated nucleoside diphosphate kinase. Mol Biol Cell.

[59] Yang TB, Poovaiah BW (2000). Molecular and biochemical evidence for the involvement of calcium/calmodulin in auxin action. J Biol Chem.

[60] Pastra-Landis SC, Evans DR, Lipscomb WN (1978). The effect of pH on the cooperative behavior of aspartate transcarbamylase from Escherichia coli. J Biol Chem.

