# Supplementary material for: Calcium/calmodulin alleviates substrate inhibition in a strawberry UDP-glucosyltransferase involved in fruit anthocyanin biosynthesis
Source: BMC Plant Biol. 2016 Sep 8;16(1):197. doi: 10.1186/s12870-016-0888-z (PMC5017016; doi:10.1186/s12870-016-0888-z)
Supplement: Additional file 1:Figure S1. — Amino acid sequence alignment of FvUGT1 and six other plant UGTs. Color and intensity change indicates the differences in the level of conservation. Dark red and dark blue represent the highest and lowest conserved levels, respectively. α-helices and β-strands are marked by black lines. The putative secondary plant glycosyltransferase (PSPG) motif is underlined by a red line and 10 conserved sugar donor interacting residues of the PSPG motif are marked with black solid triangles. The putative calmodulin-binding region in FvUGT1 is indicated by a black open box. The GenBank accession numbers or sources of proteins are FvUGT1 (KP165417; F. vesca), VtGT1 (AAB81682; grape), FaGT1 (AAU09442; F. × ananassa), Ct3GT (BAF49297; C. ternatea), MtUGT78G1 (A6XNC6.1; M. truncatula), AtUGT72B1 (Q9M156.1; A. thialiana), MtUGT71G1 (AAW56092.1; M. truncatula), and MtUGT85H2 (2PQ6_A; M. truncatula). (PDF 787 kb) [file 12870_2016_888_MOESM1_ESM.pdf]

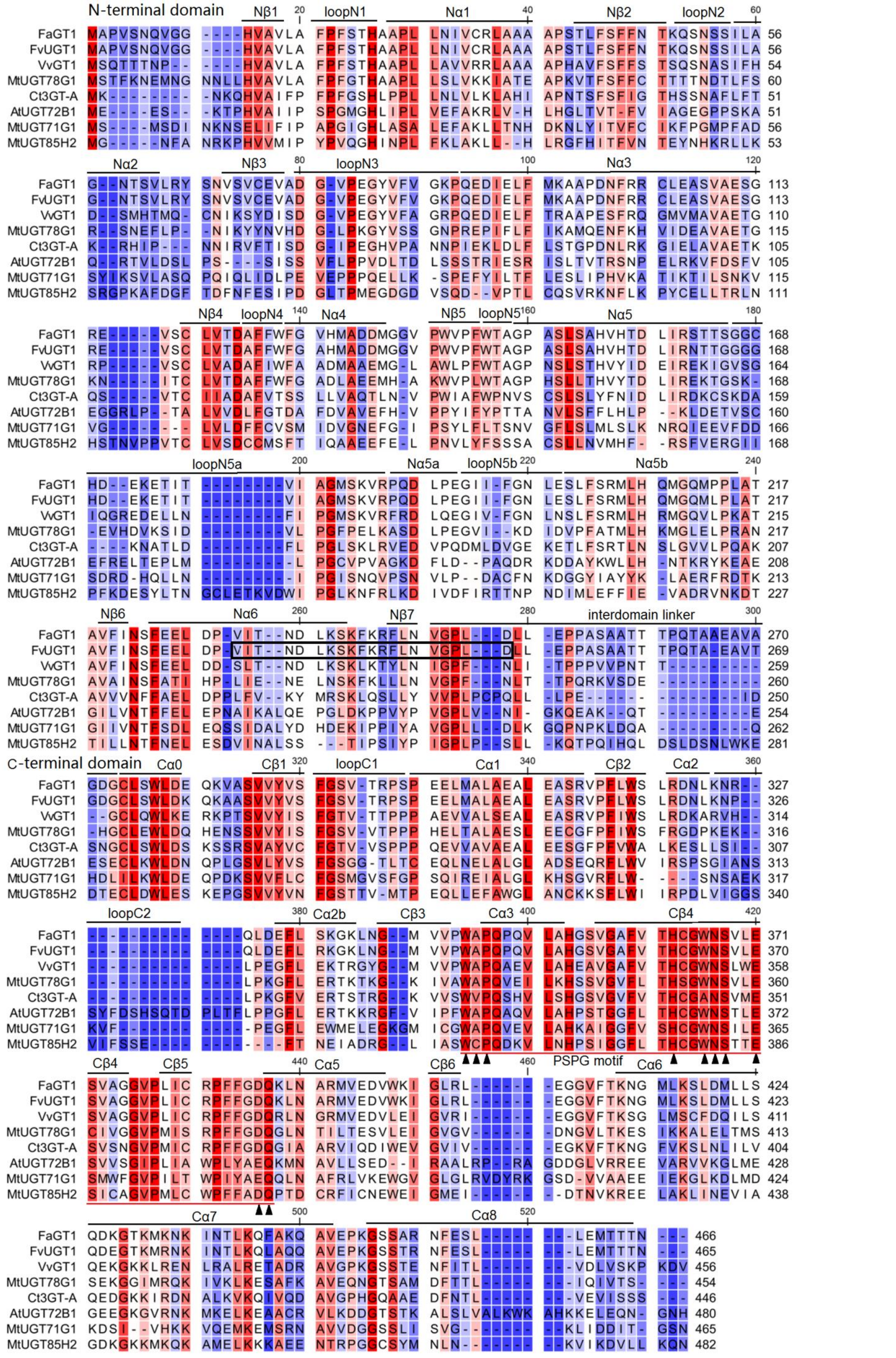
N-terminal domain
Nβ1
loopN1
Nα1
Nβ2
loopN2
Nα2
Nβ3
loopN3
Nα3
Nβ4
loopN4
Nα4
Nβ5
loopN5
Nα5
loopN5a
Nα5a
loopN5b
Nα5b
Nβ6
Nα6
Nβ7
interdomain linker
C-terminal domain
Cα0
Cβ1
loopC1
Cα1
Cβ2
Cα2
loopC2
Cα2b
Cβ3
Cα3
Cβ4
Cβ5
Cα5
Cβ6
PSPG motif
Cα6
Cα7
Cα8
FaGT1
FvUGT1
VvGT1
MtUGT78G1
Ct3GT-A
AtUGT72B1
MtUGT71G1
MtUGT85H2
